# Giant Dielectric Permittivity in Ferroelectric Thin Films: Domain Wall Ping Pong

**DOI:** 10.1038/srep14618

**Published:** 2015-10-06

**Authors:** An Quan Jiang, Xiang Jian Meng, David Wei Zhang, Min Hyuk Park, Sijung Yoo, Yu Jin Kim, James F. Scott, Cheol Seong Hwang

**Affiliations:** 1 State Key Laboratory of ASIC & System, School of Microelectronics, Fudan University, Shanghai 200433, China; 2 National Laboratory for Infrared Physics, Shanghai Institute of Technical Physics, Chinese Academy of Sciences, Shanghai 200083, China; 3 Department of Materials Science and Engineering and Inter-university Semiconductor Research Center, Seoul National University, Seoul 151-744, Korea; 4 School of Chemistry and School of Physics, St. Andrews Univ., St. Andrews, U.K. KY16 9ST

## Abstract

The dielectric permittivity in ferroelectric thin films is generally orders of magnitude smaller than in their bulk. Here, we discover a way of increasing dielectric constants in ferroelectric thin films by ca. 500% by synchronizing the pulsed switching fields with the intrinsic switching time (nucleation of domain plus forward growth from cathode to anode). In a 170-nm lead zirconate titanate thin film with an average grain size of 850 nm this produces a dielectric constant of 8200 with the maximum nucleus density of 3.8 μm^−2^, which is one to three orders of magnitude higher than in other dielectric thin films. This permits smaller capacitors in memory devices and is a step forward in making ferroelectric domain-engineered nano-electronics.

High-dielectric ferroelectric thin-films are of great importance for nanoelectronics, where their capacitance should be maximum to reduce size and power consumption. The capacitance can be enhanced by making the films thinner, but that is limited by breakdown. An attractive alternative would be to increase the dielectric constant. An increase of an order of magnitude would permit a smaller “footprint” of areal size on the chip; since 90% of the area of such a chip is capacitor, with smaller resistors and transistors taking up only ca. 10%, this would permit a 90% overall size reduction. Lead zirconate titanate (PZT) thin films could be a viable option for such dielectric material having a very high dielectric constant. In this work, the enhancement of dielectric constant in ferroelectric PZT thin films from a normal value of ca. 800 to 8,200 is reported. This increase was realized via ac-voltage drive synchronized to the anode-cathode transit time for domain wall motion. By reversing the applied electric field just before the nucleated reverse domains penetrate through interfacial electrode-dielectric (“dead”) layer to reach the opposite electrode^1^, degradation of the dielectric response was prevented. The phenomenon is analogous to volleying a tennis or ping-pong ball before it strikes the opposite surface.

Classic ferroelectric oxide films provide large ionic displacements of individual atoms down to the atomic layer thickness, for example, ~2.4 nm for SrRuO_3_/BaTiO_3_/SrRuO_3_ sandwiches, and the related functionalities in these devices can be achieved in the ns-ps time scale as their physical dimensions shrink down into the nanometer scale^2^,^3^,^4^,^5^,^6^. In principle, the very large ionic polarization charges in ferroelectrics can create a huge dielectric response with frequencies of up to several GHz if the polarization (*P*_*f*_) can be reversibly switched to follow the external stimuli of an alternating-current (AC) field (*E*_*f*_). However, the experimental value of the dielectric constant is always much smaller than the expected value (*ε* = *dP*_*f*_/ε_0_*dE*_*f*_, where *ε*_*0*_ is the vacuum permittivity) because much of the polarization charge does not follow the small oscillating AC field. Therefore, these effects have severely hampered the applications of ferroelectrics to memory devices, miniaturized sensors, actuators, phase shift antenna arrays, and energy harvesting systems^7^,^8^.

It has also been known since the 1980s^9^,^10^,^11^,^12^,^13^,^14^,^15^,^16^,^17^,^18^ that domain walls can contribute to the dielectric constant in a different way. That is walls can oscillate laterally and reversibly even without complete ferroelectric domain switching, and, thus, add to the dielectric susceptibility^19^,^20^. In the present study these early ideas are extended to the longitudinal oscillation of domains that do not quite extend from a cathode to an anode. This huge dielectric response arising from the domain oscillation can occur at temperatures below the Curie point, which is completely different from the large enhancement in dielectric constant near the ferroelectric-paraelectric phase transition of several ferroelectric materials, such as the epitaxial (Ba,Sr)TiO_3_ thin films^21^.

## Results

### Principle of nucleating domain oscillation

**Figure 1:**
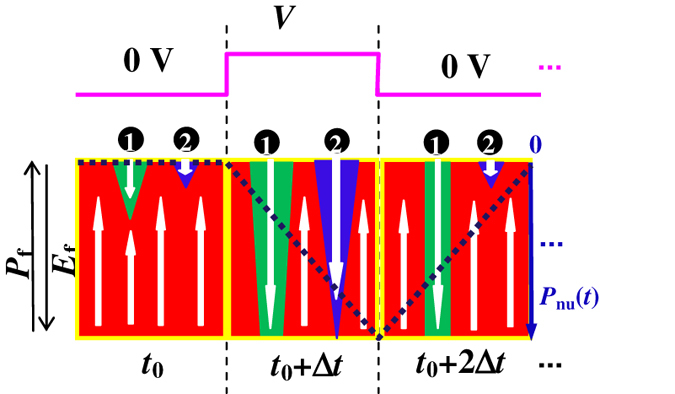
As the source voltage was increased from 0 V to *V* across the pre-poled capacitor with an *E*_*f*_ that was antiparallel to *P*_*f*_ at *t*_0_, the two reverse domain nuclei 1 and 2 that stemmed from the interface began to grow at *t*_0_ + Δ*t.* As the voltage dropped back to 0 V at *t*_0_ + 2Δ*t*, the non-penetrating Domain 2 within the film thickness contracted to its previous state, in contrast to the irreversibly penetrated Domain 1. The Domain 2 motion after *t*_0_ + 2Δ*t* reversibly followed the external AC pulse field and generated the polarization *P*_*nu*_ shown by the thick dotted line.

In BaTiO_3_ single crystals with hetero-valence impurities a large nonlinear electrostriction is generated during 90° domain switching^22^; a restoring force arises from temporarily uncompensated charged defects. In ferroelectric thin films the restoring force can originate from the temporarily uncompensated charges of the moving fronts of domain walls^23^,^24^,^25^. In the present work, this basic idea was used to maximize the dielectric constant of PZT thin films of geometry and electrode materials suitable for real nanocapacitor devices, and an increase in dielectric constant from 800 to 8,200 was obtained. The geometry of the problem is simple, but the algebraic details complicated; so the algebraic is separated into sections in the on-line Supplementary Information (on-line SI). The complex equations are unfortunately required to obtain true values of dielectric constant in a device with electrodes, interfacial regions, forward- and sideways-growth of domains, reverse switching voltages, etc. The key requirement is that the domain wall velocity distribution must be narrow. It is emphasized at the outset that there are no adjustable parameters in this model; all numerical values are highly reproducible on numerous samples and agree with independent literature values. It is important for readers to keep in mind several simple things about ferroelectric switching: (a) It is almost 100% inhomogeneous nucleation (no spinodal decomposition), generally at the electrode-dielectric interface; (b) the walls move as needle-like shapes from cathode to anode (or vice-versa) at subsonic speeds with little variation in speed; (c) therefore, their transit time can be synchronized to the applied AC field just in time to reverse their direction and prevent penetration into the opposite electrode-dielectric interface. Figure 1 schematically shows this idea, which shows the changes in the polarization states of a ferroelectric film when a short anti-parallel voltage pulse (*V*) is applied. This figure implicitly assumes a single crystalline film, but the same model can be applied to coarse-grained polycrystalline films when the interference effect from the presence of grain boundaries is weak. It is assumed that the down-polarized domains at time *t*_0_ have residual back-switched clusters or “nuclei” even in the upward pre-poled state (left panel in Fig. 1). When an applied voltage pulse, *V*, is suddenly applied at a certain time (*t*_*0*_ + *Δt*), Nucleus 1 is assumed to grow rapidly and to form a fully switched domain during the interval time of *Δt*, whereas Nucleus 2 is still penetrating the film thickness (middle panel in Fig. 1). When *V* is removed at *t*_*0*_ + 2*Δt*, Domain (Nucleus) 1 remains unchanged, but Domain (Nucleus) 2 shrinks back quickly and releases the polarization charge, *P*_*nu*_ (right panel in Fig. 1). Therefore, the ferroelectric polarization charges of Domain 1 cannot contribute to the discharges, but those of Domain 2 do so when the discharging charges were monitored after *t* = *t*_*0*_ + 2*Δt*^5^.

Experimental evidence of the reversible nucleating domain growth and the subsequent sideways wall motion has been found in the cross-sectional transmission electron microscopy observation of non-penetrating triangular domains in epitaxial (001) Pb(Zr_0.2_Ti_0.8_)O_3_ thin films, where the embedded *in-situ* piezoresponse force microscopy (PFM) probes induced triangular domain nucleation with a wall that was inclined by 55° toward the substrate^26^. In such cases the non-penetrated domains indeed shrank back rapidly when the switching voltage was turned off.

### Time of domain forward growth

Under an AC driving field, the nucleating domains can oscillate longitudinally and thus contribute to the nonlinear capacitance through the periodic domain expansion and contraction. To achieve this goal, the contribution of *P*_*nu*_ to the achievable *ε* must be maximized by precisely controlling the domain oscillations and transit times within the film thickness. First, the critical time *Δt* needed for the nucleated reverse domain to touch the opposite electrode at different nucleating current densities (or *V*) must be estimated. This can be done by understanding that *Δt* is the time needed for the switching current flows to induce *P*_*nu*_, with the current density of^6^


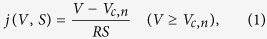


where *V*_c,n_ and *S* are the coercive voltage for domain nucleation and the electrode area, respectively. Thus:


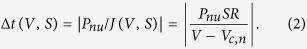


**Figure 2:**
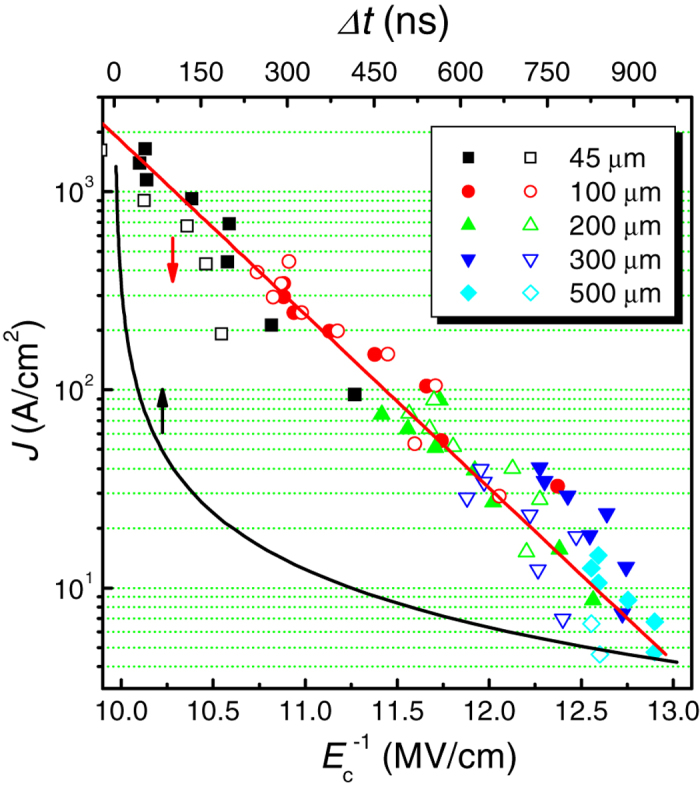
The closed and open symbols show the switching current density as a function of the inverse coercive field of the PZT in capacitors with different sizes for the forward expansion and sideways wall motion of the nucleating domains, respectively. The data can be fitted by a red solid line, according to Merz’s law. The black solid line shows the domain expansion time as a function of the current density for the nucleating domains to touch the opposite electrode.

*J* was measured as a function of inverse of coercive field 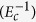, and the results for the capacitors with various sizes are summarized in Fig. 2. For this work, Pt/Pb(Zr_0.4_Ti_0.6_)O_3_(PZT)/Pt polycrystalline thin film capacitors with a 170-nm PZT thickness and an average PZT grain size of ~800 nm were deposited on TiO_x_/SiO_2_/Si substrates (on-line SI Part **D**: Fig. S8a) using a sol-gel processing technique. Then the films were patterned into discrete square capacitors (see Methods). The data in Fig. 2 are well described by Merz’s exponential law^6^, 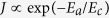, as shown by the (red) solid line. The fitting of the experimental data to Merz’s law gave an activation field *E*_*a*_ = 2.0 MV/cm, which is consistent with a previous report^27^. From the result |*P*_*nu*_| = 4.1 μC/cm^2^, which will be discussed in detail below, *Δt* can be calculated, as shown by the (black) solid line in Fig. 2 using Eq. (2). This is the approximate time for the nucleating domains to touch the opposite electrode at each *J* (or *V*).

### Giant dielectric permittivity characterized from a delta-pulse technique

Using the calculated *J*(*V*, *S*) and *Δt*(*V*, *S*) functions, the ferroelectric capacitance-voltage (*C*_*f*_–*V*_*f*_) loops for a 100 × 100-μm^2^-area capacitor can be directly estimated using a delta pulse technique (on-line SI Part A, Figs S1–S5). With this technique *V* was applied to a pre-poled capacitor with a −6 V voltage for 600 ns, with an increasing *V* value from −5 to 5 V in 0.05 V steps (Δ*V*_step_). At each voltage the nonlinear capacitance was measured from the difference between the two discharging capacitor charges at the two voltages of *V*_*f*_ + Δ*V*_*f*_ and *V*_*f*_ , as follows:


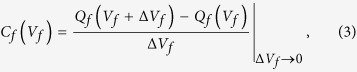


where *Q*_*f*_ is the capacitor-accumulated charge per area and *V*_*f*_ is the voltage across the ferroelectric layer under *V* in the RC circuit. When the pulse time is short enough to keep the domains within the film thickness at a given *V*_*f*_, the capacitance is dominated by the reversibly oscillating domains. However, this type of measurement does not necessarily exclude the contribution from the polarization switching charge when the sweeping voltage changes by *ΔV*_step_, especially when the polarity of *V*_*f*_ becomes opposite to the pre-poling voltage direction. To alleviate this problem the capacitor was repeatedly stressed at each value of *V*_*f*_ with a unipolar pulse width of 250 ns for 70 cycles, where the net voltage drop across the film is


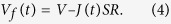


**Figure 3:**
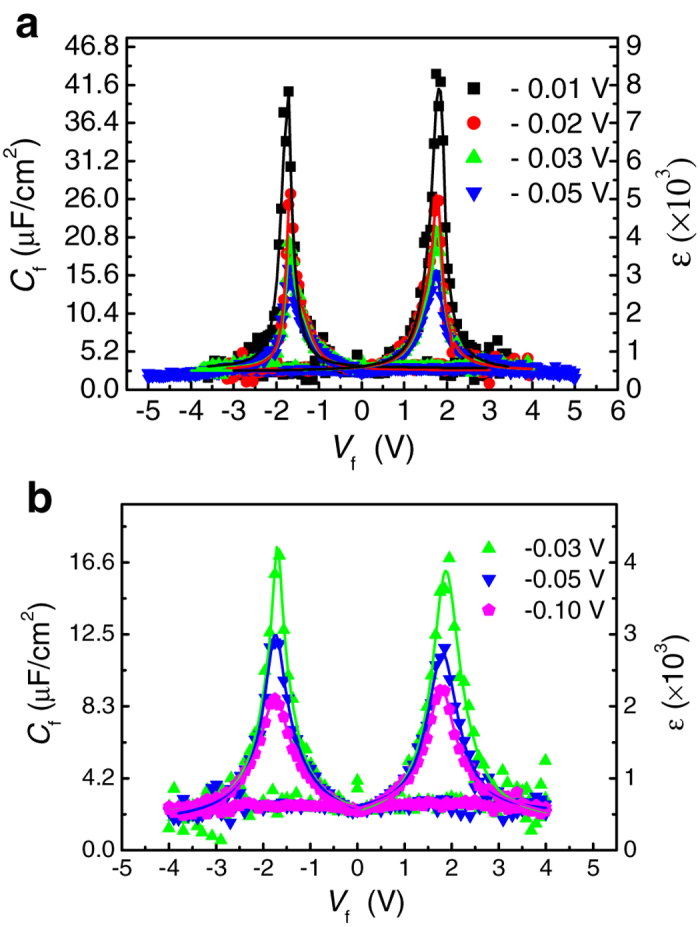
(**a**) *C*_*f*_ –*V*_*f*_ loops for a 10^4^ μm^2^-area capacitor under different Δ*V*_*f*_ values, with a stressing time of 250 ns at each voltage. (**b**) *C*_*f*_ –*V*_*f*_ loops for a 2 × 10^3^ μm^2^-area capacitor under different Δ*V*_*f*_ values with a stressing time of 60 ns at each voltage. The solid lines are the guides for the eyes.

This was proven sufficient to remove the current contribution from irreversible polarization switching between *V* and *V* − *ΔV*_step_ (*ΔV*_step_ = 0.05 V, on-line SI Part **A**: Fig. S2a). After this pretreatment, *Q*_*f*_(*V*_*f*_) and *Q*_*f*_ (*V*_*f*_ + Δ*V*_*f*_) were measured sequentially by superimposing Δ*V* < 0 above *V* (on-line SI Part **A**: Fig. S2b), where Δ*V*_*f*_ is the delta voltage sensed by the ferroelectric layer only. Here, a pulse period of 250 ns was adopted at each *V*, when the capacitor was electrically charged for the first 70 ns; and the discharging current induced by Δ*V*_*f*_ < 0 was estimated during the subsequent 180 ns. The pulse period is far larger than the circuit RC time constant of 37.2 ns before the involvement of nucleus domain oscillation (on-line SI Part D, Fig. S9a). From this method the charge difference between *Q*_*f*_(*V*_*f*_ + Δ*V*_*f*_) and *Q*_*f*_(*V*_*f*_) was estimated; this was attributed to the shrinking domain charges (*P*_*nu*_) and normal dielectric capacitor charges of both unswitched (or yet-to-be-switched) and switched regions. Finally, the complete *C*_*f*_ –*V*_*f*_ loops with increasing and later decreasing *V*_*f*_ values (−5 V → 5 V → −5 V) were measured under the different stimulating AC voltages of *ΔV*_*f*_ (from −0.01 to −0.05 V). The results are summarized in Fig. 3a. It is important to note that *ε* reached a maximum of 8,200 when Δ*V*_*f*_ = −0.01 V, which is the largest dielectric permittivity ever reported from ferroelectric thin films (with the exception of the boundary layer supercapacitors^28^). During the partial domain switching, the ferroelastic domain-wall contribution to the dielectric response should also be accounted for^8^,^9^,^10^,^11^,^12^,^13^,^14^,^15^,^16^,^17^. Recent report by Karthik, *et al.*, has shown that this permittivity is ~150–250^18^. Considering this effect, *ε* *~* 8,000 should be ascribed purely to the dielectric response of the oscillating domains. It is different from other contributions by random fields, compositional and mesostructural heterogeneities near phase transition (Curie) temperatures and/or morphotropic phase boundaries, which should be below the order of 630 estimated from the measurements in a long mismatched time of Δ*t* = 100 ms (on-line SI Part **A**: Fig. S4a). When Δ*V*_*f*_ decreases from −0.01 to −0.05 V, *ε* decreases by more than a factor of 3. This is presumably due to the large *P*_*nu*_(*V*_*f*_) nonlinearity (or the wide *P*_*nu*_ distribution) near the coercive voltage (*V*_c,n_) for reverse domain nucleation (on-line SI Part **A**: Fig. S4c). This can be distinguished from the nonlinear enhancement of the pinning wall vibrations with increasing voltage amplitude (on-line SI Part **A**: Figs S4b and 4c and refs 29 and 30). *P*_*nu*_ showed sharp peaks when *V*_*f*_ = *V*_c,n_. Therefore, the average discharge per Δ*V*_*f*_ decreases as |Δ*V*_*f*_| increases near *V*_c,n_ (on-line SI Part **B**: Fig. S7a). Once the voltage stressing time matches domain nucleation time, the dielectric permittivity shows a maximum, as evidenced from time dependence of dielectric permittivity under different stressing voltages close to *V*_c,n_ (on-line SI Part **F**: Figs S11a and b). Otherwise, the dielectric permittivity decays rapidly with mismatched time and voltage.

Alternatively, the domain nucleation time can be adjusted through the change of the capacitor area according to Eq. (2). Figure 3b shows *C*_*f*_ –*V*_*f*_ loops at different Δ*V*_*f*_ values with a dc stressing time of 60 nm for a capacitor area of 2 × 10^3^ μm^2^. This dc stressing time is far larger than the circuit RC time constant of 9.2 ns before the involvement of nucleus domain oscillation (on-line SI Part D, Fig. S9b). The largest dielectric permittivity at Δ*V*_*f*_ = −0.03 V is ~4000, quite comparable to the value in Fig. 3a at the same stimulating voltage. This value could be even higher when the Δ*V*_*f*_ was −0.01 V, but this could not be confirmed due to the higher noise to signal at such small Δ*V*_*f*_ and *S* (on-line SI Part **D**: Fig. S9c).

### Nucleus domain density

**Figure 4:**
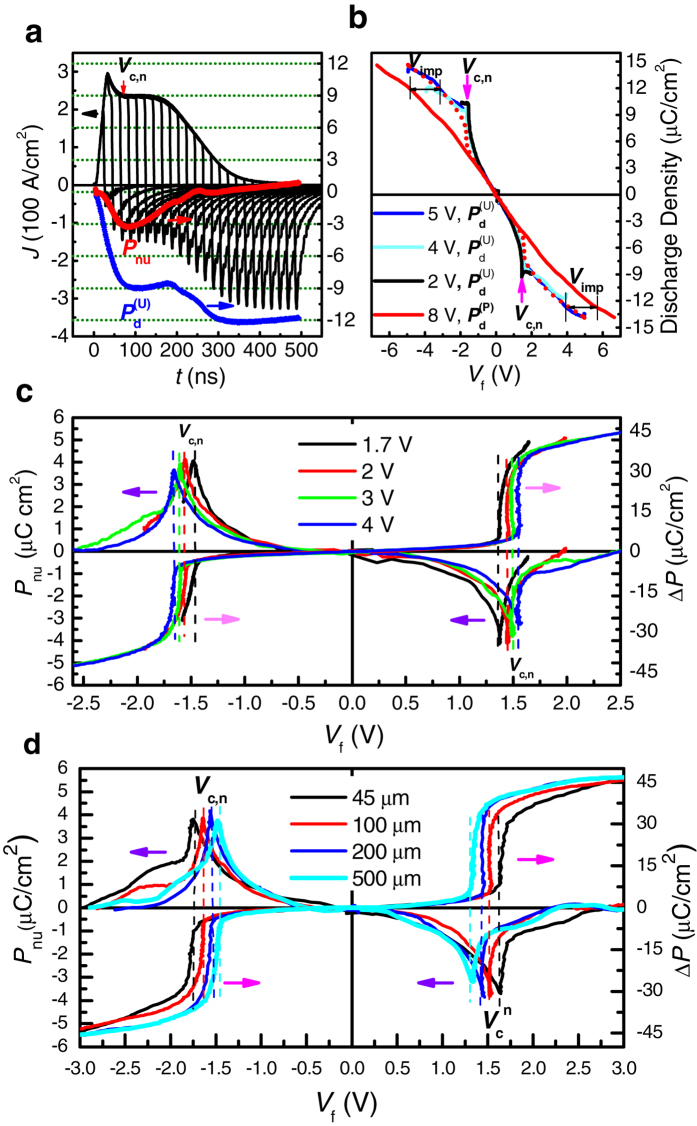
(**a**) Domain switching and discharging current transients at different pulse widths under *V* = 4 V for a 10^4^ μm^2^-area capacitor, wherein the solid-colored lines show *P*_d_^(AP)^ and *P*_*nu*_ as functions of the pulse width. (**b**) Voltage dependence of the capacitor discharging charge density under different *V* values, with *E*_*f*_ parallel/antiparallel to *P*_*f*_, where the dotted line shows the short-time imprint effect on the shift 
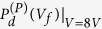
 and the irreversible polarization reversal. The reversible and irreversible polarizations are separated under (c) different *V* values for a 10^4^ μm^2^-area capacitor and (d) different capacitor sizes at *V* = 3.5 V.

Although the large *ε* of ~8,200 was measured experimentally, it should be proven that such a large *ε* results mainly from *P*_*nu*_ of the reversibly switched domains. Figure 4a shows an example of how the total domain switching and discharging currents, as well as *V*_*f*_, can be measured in a constant-voltage pulse switching test. In this case a constant voltage of *V* = 4 V was programmed into the voltage pulse generator, and the pulse width (time) τ was increased in a stepwise manner from 5 to 500 ns which were applied to the top electrode of a 100 × 100 μm^2^-area capacitor (Fig. 4a). The current density transients [*J*(*t*)] as well as their transformation into *P*_*f*_-*V*_*f*_ hysteresis loops (on-line SI Part **B**: Figs S6a–6c) were measured using an oscilloscope, which was serially connected to the sample with the total internal resistance of *R* = 100 Ω at ambient temperature. 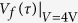 at the pulse width *t* = τ can be obtained from Eq. (4). From *J*(*t*) the capacitor discharge and domain switching charge can be calculated as functions of *V*_*f*_. To calculate the discharge free from the influence of the irreversible domain switching charge, the dissipating current after *t* > τ was integrated, and the results were plotted as functions of *V*_*f*_ for different *V* values, as shown in Fig. 4b. Such measurements of the discharge density were repeated for the *V* values that were anti-parallel 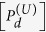 and parallel 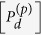 to the previous poling direction of the PZT capacitor. The switched polarization (Δ*P*) can also be estimated from the integration of the entire *J* (*t*) curve (0 < *t* < ∞) for each *V*_*f*_ (i.e., for a given *V* pulse width), as it comprises domain switching and capacitor charging/discharging. The results are shown in Fig. 4c,d for different *V* values (size = 10^4^ μm^2^) and sizes (*V* = 3.5 V). In Fig. 4a the rapid increase and peaked behavior in *J*(*t*) up to ~50 ns are attributed to pure capacitor charging and reverse domain nucleation. The subsequent constant *J*(*t*) up to ~200 ns corresponds to the sideways wall motion of the domain growth (to be discussed later). Then, the current decays exponentially with time as the domain switching is completed, and *V*_f_ eventually reaches *V*. Also shown in Fig. 4a are the variations in 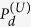 and *P*_*nu*_, as functions of time. When the same measurement was performed with a voltage whose polarity was parallel to the pre-poling direction, the *J*(*t*) and 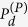 values were obtained for the non-switching case. In Fig. 4b it is noted that 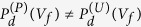, which is inconsistent with the usual expectation that the two must have identical values (i.e., the discharge estimated in this way must be a reversible dielectric displacement charge at a given *V*_*f*_ if there is no contribution from other factors). There were two more critical observations: (1) all the 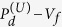 plots at *V* = 2–5 V showed sharp peaks near the coercive voltages for the reverse domain nucleation (*V*_c,n_), as indicated by the (pink) arrows in Fig. 4b, which are not seen in the 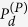
–*V*_*f*_ graphs; and (2) 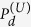 (*V*_*f*_) equals 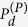 (*V*_*f*_  + *V*_imp_) for |*V*_*f*_ | > |*V*_c,n_|, which suggests that the difference between 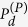 and 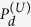 could be attributed to the presence of the uncompensated domain wall charges that were driven back by *V*_imp_. The sharp peaks at *V*_c,n_ in Fig. 4b provide unambiguous evidence that the uncompensated domain wall charges and imprint voltage indeed shrank the nucleating domains when *V* was turned off. This means that some parts of the domain inversion were not completed throughout the film thickness during the period of the application of *V*. Therefore, the full nucleation charge *P*_nu_, which was discharged when *V* was turned off, can be defined. This corresponds to the discharge related to the rapidly shrinking reverse domains, as represented by Domain 2 in Fig. 1. The values of *V*_c,n_ that differ from the coercive voltage (*V*_c,s_) for sideways wall motion can be estimated from the sharp peak position in Fig. 4b. *V*_c,s_ corresponds to the voltage at which half of the irreversible domain reversal is completed, which is usually regarded as the coercive voltage of a ferroelectric capacitor in the measurement of the polarization-voltage (*P*_*f*_ –*V*_*f*_) hysteresis loop. The presence of a hump near *t* ~ 70 ns in the 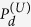
–*t* plot in Fig. 4a suggests that the nucleation occurred before the onset of the sideways wall motion (current plateau in *t* ~ 80–150 ns). It is quite natural that these *P*_*nu*_ peaks were not observed in the 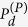
–*V*_*f*_ graphs, as there was no reverse domain nucleation and growth. The imprint field is believed to be built in by the interfacial trapped charges during the pre-poling^25^, and to become temporarily unscreened immediately after the reverse domain nucleation.

To estimate quantitatively *P*_*nu*_ and to understand its influence on *ε* of the PZT layer, the following points were considered: It was noted that 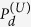 (*V*_*f*_) in Fig. 4a should have three components: (1) the charge density related to the reversible shrinking domains [Domain 2 in Fig. 1, *P*_nu_(*V*_*f*_)]; (2) the charge density related to the non-switched (or yet-to-be-switched) regions [the red region in Fig. 1, 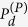 (*V*_*f*_)]; and (3) the charge density related to the irreversibly switched regions [Domain 1 in Fig. 1, 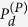 (*V*_*f*_  + *V*_imp_)]. The volume fraction of the switched domain, *x*, at a given *V*_*f*_ can be estimated from 
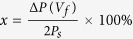
, where *P*_*s*_ is the saturation polarization inferred from the saturated *P*_*f*_ –*V*_*f*_ hysteresis loop (Fig. S7b). Therefore, 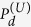 (*V*_*f*_) is the sum of *P*_*nu*_(*V*_*f*_), [1 − *x*(*V*_*f*_)]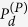 (*V*_*f*_) and *x*(*V*_*f*_)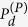 (*V*_*f*_  + *V*_imp_). 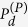 (*V*_*f*_) could be represented by 
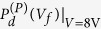
, as shown in Fig. 4b, since 8 V of *V* is large enough to induce all the needed charging. Δ*P*(*V*_*f*_), which is needed to calculate *x* at each *V*_*f*_ , is already given in Fig. 4c,d. Therefore,


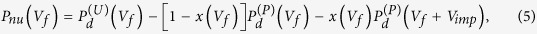


and *P*_*nu*_ can be calculated as a function of *V*_*f*_ for different *V* and *S* values (Fig. 4c,d). Each *P*_*nu*_(*V*_*f*_) curve has a sharp peak near *V*_*f*_ that coincides with the onset of an abrupt increase in |Δ*P*|. When *V*_*f*_ at which half of the |Δ*P*| increase is obtained is taken as *V*_c,s_ for the domain sideways wall motion, |*V*_*f*_| for the peaked *P*_*nu*_ is slightly lower than |*V*_c,s_|. Such *V*_*f*_ for the peaked *P*_*nu*_ can define the coercive voltage for reverse domain nucleation (*V*_c,n_): it is notable that |*V*_c,n_| is always slightly lower than |*V*_c,s_| for sideways wall motion for different *V* and *S* values (Fig. 4c,d). Theoretically *ε* of the ferroelectric layer can be calculated from the *P*_*nu*_–*V*_*f*_ and 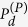
–*V*_*f*_ plots in Fig. 4b–d using the formula


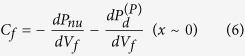


for |*V*_*f*_| ≤ |*V*_c,n_|. The *C*_*f*_ –*V*_*f*_ graphs are shown in Fig. S3 of on-line SI Part **A**, and they are reasonably consistent with those in Fig. 3a. In addition, the maximum |*P*_nu_| = 4.1 μC/cm^2^ was quantitatively evaluated. It is almost independent of the *V* and *S* values (the slight drop in the largest current density was due to the limited data collection speed of the measurement setup). This value slightly increases up to 4.4 μC/cm^2^ at 77.6 K (on-line SI Part **C**: Fig. S8d).

From the assumed conical shape of a non-penetrating domain with an inclined wall angle of α = 55° 26, we estimated the maximum nucleus density of *N* = 3.8 μm^−2^ at *V*_c,n_ from the formula of


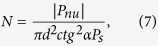


where *d* is the film thickness. This is more than one-order-of-magnitude lower than the highest concentrations of the reported interfacial point defects (10^2^ μm^−2^)^14^, which suggests that nucleation of reverse domains may occur at sites with any extended defects. In this regard, unpublished work by Y. Ivry (Cambridge) showing effects of screw dislocations in PZT is known to the authors. The maximum nucleus density of ~3.8 μm^−2^ from this experiment with macroscopic capacitors (one or two effective nucleation sites per grain at *V*_c,n_ where the average grain size is 850 nm) is comparable to the values of 4.4 μm^−2^ (area: 1.5 × 1.5 μm^2^) in ref. 31 and 3.4 μm^−2^ (area: 3 × 3 μm^2^) in ref. 32 from microscopic PFM images of switched regions covered by a thin layer of top electrode. In those works, the numbers of independent penetrating domain areas across the film thickness were counted assuming that there is no reverse domain nucleation within the film thickness. The estimated nucleation density also confirms with the ultrafast switching experiments by Grigoriev, *et al.*, using focused X-ray microdiffraction^33^.

## Discussion

The polarization oscillations of the non-penetrating domains within the thickness of ferroelectric PZT thin films (170 nm) were observed under a well-controlled AC pulse field. With the help of the short-time imprint effect, the maximum reversible polarization of ~4.1 μC/cm^2^ at *V*_c,n_ due to the non-penetrating reverse domain nucleation was quantitatively estimated. The restoring force (imprint voltage effect) was large enough to shrink the nucleating domains when the anti-parallel excitatory voltage was removed, which opens the door for the development of new domain engineering. A huge dielectric permittivity (~8,200) from the periodic domain oscillations was estimated, which was free from the interfacial “dead-layer” effects even in ultra-thin films. This longitudinal oscillation differs from the transverse oscillation of fully penetrating walls^9^,^10^,^11^,^12^,^13^,^14^,^15^,^16^,^17^,^18^,^19^,^20^. This mechanism was shown to be operative over a wide range of temperature (77.6 K–300 K) and switching time (100 ns–5 ms). These oscillators should be sensitive to the weak disturbance of external acoustic, thermal and mechanical fields, which suggests the development of new concepts and designs of sensors, actuators and other electronic devices with improved sensitivity, in addition to nanocapacitors with an extremely large stored charge density. This study is analogous to the ‘volleying’ of magnetic domain walls with synchronized pulsed AC magnetic fields in the magnetic race-track memory^34^,^35^,^36^.

## Methods

### Sample preparation

Polycrystalline Pb(Zr_0.4_Ti_0.6_)O_3_ thin films were synthesized through sol-gel spin coating on Pt/TiO_x_/SiO_2_/Si substrates with a methoxyethanol-based precursor solution^11^. The as-deposited layers that were initially thermal-soaked at 300 °C for 3 min and were finally crystallized at 750 °C for 10 min with 130-nm and 170-nm film thicknesses. The average grain size (~800 nm) of this PZT film was estimated from either scanning electron microscopy or transmission electron microscopy (JEOL 3000F) observation. Pt and hybrid IrO_2_-Pt electrodes were sputtered on the films as the bottom and top electrodes (See Fig. S8a of on-line SI). After the films were photo-lithographically patterned and dry-etched into discrete square capacitors with 45–500 μm side lengths, they were re-annealed at 700 °C for 10 min. To reduce the *P*_*nu*_(*V*_*f*_) distribution, all the samples were first stressed at ±6 V with suitable bipolar pulse widths for 10^4^ cycles and then aged at ambient temperature for more than a week, to establish the same imprint history for all the random domains. The electrical measurements were carried out at both ambient and cryogenic (77.6 K) temperatures.

### Electrical setup

To measure the domain switching performance, square voltage pulses with rise times of 20 ns and fall times of 2 ns were supplied using two-channel Agilent 81110A and 81150A pulse generators. The transient currents across in-series resistors of R = 100 Ω, which is the sum of internal resistances of pulse generator and oscilloscope, were monitored using LeCroy WR6200A and HDO6054 oscilloscopes in 8- and 12-bit voltage resolutions with bandwidths of 2 GHz and 500 MHz, respectively. For comparison, the capacitance vs. voltage loops at long voltage sweep times were collected using an HP 4194A impedance analyzer.

## Additional Information

**How to cite this article**: Quan Jiang, A. *et al.* Giant Dielectric Permittivity in Ferroelectric Thin Films: Domain Wall Ping Pong. *Sci. Rep.* **5**, 14618; doi: 10.1038/srep14618 (2015).

## Supplementary Material

Supplementary Information

## References

[b1] Jiang A. Q., Lee H. J., Kim G. H. & Hwang C. S. The inlaid Al_2_O_3_ tunnel switch for ultra-thin ferroelectric films. Adv. Mater. 21, 2870–2875 (2009).

[b2] Junquera J. & Ghosez P. Critical thickness for ferroelectricity in perovskite ultrathin films. Nature 422, 506–509 (2003). 12673246 10.1038/nature01501

[b3] Warusawithana M. P. *et al.* A ferroelectric oxide made directly on silicon. Science 324, 367–370 (2009). 19372426 10.1126/science.1169678

[b4] Li J. *et al.* Ultrafast polarization switching in thin-film ferroelectrics. Appl. Phys. Lett. 84, 1174–1176 (2004).

[b5] Rana D. S. *et al.* Understanding the Nature of Ultrafast Polarization Dynamics of Ferroelectric Memory in the Multiferroic BiFeO_3_. Adv. Mater. 21, 2881–2885 (2009).

[b6] Jiang A. Q., Lee H. J., Hwang C. S. & Scott J. F. Sub-picosecond processes of ferroelectric domain switching from field and temperature experiments. Adv. Funct. Mater. 22, 192–199 (2012).

[b7] Baek S. H. *et al.* Domain Dynamics During ferroelectric Switching. Science 334, 968–971 (2011). 22096196 10.1126/science.1206980

[b8] Trolier-McKinstry S. *et al.* Designing piezoelectric films for micro electromechanical systems. IEEE Trans. Ultrason. Ferroelectr. Freq. Control 58, 1782–1792 (2011). 21937309 10.1109/TUFFC.2011.2015

[b9] Sidorkin A. S. & Fedosov V. N. Quasi-elastic displacement of domain displacements of domain boundaries in ferroelectrics. Fiz. Tverd. Tela 18, 1661–1668 (1976).

[b10] Darinskii B. M. & Sidorkin A. S. Domain-wall oscillations in ferroelectrics and ferroeleastics. Fiz. Tverd. Tela 29, 3–7 (1987).

[b11] Darinskii B. M. & Sidorkin A. S. Domain-wall structure near the surface of a ferroelectric. Fiz. Tverd. Tela 31, 287–289 (1989).

[b12] Sidorkin A. S. & Nesterenko L. P. Efficient mass and self-frequency of oscillations for the transmission movement of 180 degrees domain boundaries in ferroelectrics and ferroelastics. Fiz. Tverd. Tela 37, 3747–3750 (1995).

[b13] Sidorkin A. S. & Sigov A. S. Translational vibrations of domain boundaries in ferroelectrics with defects. Ferroelectrics 219, 835–841 (1998).

[b14] Pertsev N. A., Arlt G. & Zembilgotov A. G. Domain-wall and intrinsic contributions to the dielectric response of epitaxial ferroelectric films. Microelectronic Engineering 29, 135–40 (1995).

[b15] Zhang Q. M., Wang H., Kim N. & Cross L. E. Direct evaluation of domain-wall and intrinsic contributions to the direct and piezoelectric response and their temperature-dependence of lead-zirconate-titanate ceramics. J. Appl. Phys. 75, 454–459 (1994).

[b16] Pertsev N. A., Arlt G. & Zembilgotov A. G. Prediction of a giant dielectric anomaly in ultrathin polydomain ferroelectric epitaxial films. Phys. Rev. Lett. 76, 1364–1367 (1996). 10061702 10.1103/PhysRevLett.76.1364

[b17] Xu F. *et al.* Domain wall motion and its contribution to the dielectric and piezoelectric properties of lead zirconate titanate films. J. Appl. Phys. 89, 1336–1348 (2001).

[b18] Karthik J., Damodaran A. R. & Martin L. W. Effect of 90 degrees Domain Walls on the Low-Field Permittivity of PbZr_0.2_Ti_0.8_O_3_ Thin Films. Phys. Rev. Lett. 108, 167601 (2012). 22680755 10.1103/PhysRevLett.108.167601

[b19] Pakhomov A., Luk’yanchuk I. & Sidorkin A. Frequency dependence of the dielectric permittivity in ferroelectric thin films with 180 degrees domain structure. Ferroelectrics 444, 177–182 (2013).

[b20] Brierley R. T. & Littlewood P. B. Domain wall fluctuations in ferroelectrics coupled to strain. Phys. Rev. B 89, 184104 (2014).

[b21] Carlson C. M. *et al.* A. S. Large dielectric constant (ε/ε_0_ >6000) Ba_0.4_Sr_0.6_TiO_3_ thin films for high-performance microwave phase shifters. Appl. Phys. Lett. 76, 1920 (2000).

[b22] Ren X. B. Large electric-field-induced strain in ferroelectric crystals by point-defect-mediated reversible domain switching. Nat. Mater. 3, 91–94 (2004). 14716304 10.1038/nmat1051

[b23] Nelson C. T. *et al.* Domain dynamics during ferroelectric switching. Science 18, 968–971 (2011). 22096196 10.1126/science.1206980

[b24] Sharma P., McQuaid R. G. P., McGilly L. J., Gregg J. M. & Gruverman A. Nanoscale dynamics of superdomain boundaries in single-crystal BaTiO_3_ lamellae. Adv. Mater. 25, 1323–1330 (2013). 23297058 10.1002/adma.201203226

[b25] Grossmann M. *et al.* The interface screening model as origin of imprint in PbZr_x_Ti_1-x_O_3_ thin films. I. Dopant, illumination, and bias dependence. J. Appl. Phys. 92, 2680–2687 (2002).

[b26] Gao P. *et al.* Direct observations of retention failure in ferroelectric memories. Adv. Mater. 24, 1106–1110 (2012). 22331626 10.1002/adma.201103983

[b27] Shin Y.-H., Grinberg I., Chen I.-W. & Rappe A. M. Nucleation and growth mechanism of ferroelectric domain-wall motion. Nature 449, 881–884 (2007). 17922002 10.1038/nature06165

[b28] Bodeux R. *et al.* CaCu_3_Ti_4_O_12_ thin film capacitors: Evidence of the presence of a Schottky type barrier at the bottom electrode. Thin Solid Films 520, 2632–2638 (2002).

[b29] Griggio F. *et al.* Substrate Clamping Effects on Irreversible Domain Wall Dynamics in Lead Zirconate Titanate Thin Films. Phys. Rev. Lett. 108, 157604 (2012). 22587285 10.1103/PhysRevLett.108.157604

[b30] Borderon C., Renoud R., Ragheb M. & Gundel H. W. Description of the low field nonlinear dielectric properties of ferroelectric and multiferroic materials. Appl. Phys. Lett. 98, 112903 (2011).

[b31] Stolichnov I., Malin L., Colla E., Tagantsev A. K. & Setter N. Microscopic aspects of the region-by-region polarization reversal kinetics of polycrystalline ferroelectric Pb(Zr,Ti)O_3_ films. Appl. Phys. Lett. 86, 012902 (2005).

[b32] Gruverman A., Wu D. & Scott J. F. Piezoresponse force microscopy studies of switching behavior of ferroelectric capacitors on a 100-ns time scale. Phys. Rev. Lett. 100, 097601 (2008). 18352748 10.1103/PhysRevLett.100.097601

[b33] Grigoriev A. *et al.* Nanosecond domain wall dynamics in ferroelectric Pb(Zr,Ti)O_3_ thin films. Phys. Rev. Lett. 96, 187601 (2006). 16712395 10.1103/PhysRevLett.96.187601

[b34] Parkin S. S. P., Hayashi M. & Thomas L. Magnetic domain-wall racetrack memory. Science 320, 190–194 (2008). 18403702 10.1126/science.1145799

[b35] Hayashi M. *et al.* Probing vortex-core dynamics using current-induced resonant excitation of a trapped domain wall. Nat. Phys. 4, 368–372 (2008).

[b36] Hayashi M., Thomas L., Rettner C., Moriya R. & Parkin S. S. P. Dynamics of domain wall depinning driven by a combination of direct and pulsed currents. Appl. Phys. Lett. 92, 162503 (2008).

