# Supplementary material for: Giant Dielectric Permittivity in Ferroelectric Thin Films: Domain Wall Ping Pong
Source: Sci Rep. 2015 Oct 6;5:14618. doi: 10.1038/srep14618 (PMC4594126; doi:10.1038/srep14618)
Supplement: Supplementary Information [file srep14618-s1.pdf]

Supplementary Information

Giant Dielectric Permittivity in Ferroelectric Thin Films: Domain Wall Ping-Pong

An Quan Jiang[1, a], Xiang Jian Meng[2], David Wei Zhang[1], Min Hyuk Park[3], Sijung Yoo[3], Yu Jin Kim[3], James F. Scott[4,b], and Cheol Seong Hwang[3,c]

[1]*State Key Laboratory of ASIC & System, School of Microelectronics, Fudan University, Shanghai 200433, China*

[2]*National Laboratory for Infrared Physics, Shanghai Institute of Technical Physics, Chinese Academy of Sciences, Shanghai 200083, China*

[3]*Department of Materials Science and Engineering and Inter-university Semiconductor Research Center, Seoul National University, Seoul 151-744, Korea*

[4]*Department of Physics, University of Cambridge, Cambridge CB3 0HE, UK*

---

[a] E-mail: aqjiang@fudan.edu.cn
[b] E-mail: jfs32@hermes.cam.ac.uk
[c] E-mail: cheolsh@snu.ac.kr

Table of Contents

A. Differential Capacitance Characterization

B. Domain Nucleation and Wall Sideways Motion

C. Cryogenic Domain Nucleation and Imprint

D. AC Amplitude and System Noise

E. Film Topography and Dielectric Degradation

F. Synchronization of domain nucleation and pulse application

G. References

H. Table I: List of Parameters

A. Differential Capacitance Characterization

To measure the voltage dependency of the capacitor discharging charge across the ferroelectric layer under a constant applied voltage V, the voltage pulse width must be increased in a stepwise manner with time lower than the circuit RC time constant. With this increasing pulse width, the voltage, $V_f = V - JSR$, across the ferroelectric layer gradually increases from 0 to V as the capacitor charging current density J decays to zero. From this short-pulse measurement, the imprint time at each V_f can be greatly reduced with a fixed circuit RC time constant. Before the measurement, a standard linear capacitor with 50 pF nominal capacitance was used for calibrating the composed test program, and the results are shown by the open symbols in Fig. S1. The plot was almost linear, as shown by the solid line. From this linear slope, the standard capacitance ~51.1 pF was derived, in agreement with the known value of the capacitor.

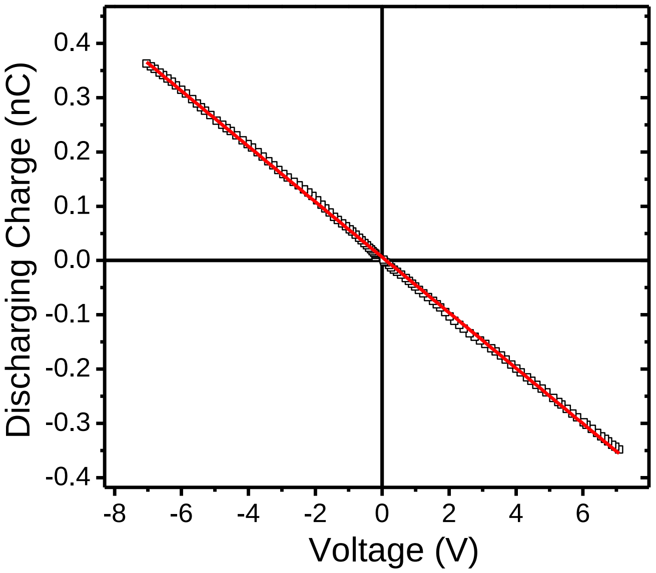

Figure S1 | Standard capacitor discharging charge. The open symbols show the voltage dependence of discharging charge for a standard linear capacitor with a nominal capacitance of 50 pF under various pulse widths, with V = 8 V. From the slope of the linear fit, a capacitance of 51.1 pF was obtained which is within 3% experimental uncertainty.

The direct measurements of the nonlinear capacitance contributed only by reversible domain motion $P_d^{(U)}$ under an alternating current (AC) field (the domain switching current contributed by the irreversible polarization $\Delta P(V_f)$ at each V_f, where ΔP always has a certain distribution with respect to V_f in a practical thin-film capacitor) must be completely removed from a delta pulse technique. In order to comply with this requirement, the following experimental procedure was adopted. After pre-poling a 10^4-μm^2-area capacitor with -6 V for 600 ns, the measurement of the switching current density at 250 ns was repeated under a unipolar pulse with a 250 ns width at a 2 MHz frequency, and with a stressing voltage either below or above the coercive voltage of $V_{c,s}$ (1.4 V vs. 3 V), as shown in Fig. S2a. It was observed that the two current densities initially decayed rapidly with the measurement number N, and that $J(N)$ had the following form:

$$J\left(N\right)=J^{(re)}+J^{(ir)}/N, \qquad \text{(S1)}$$

where $J^{(\mathrm{re})}$ and $J^{(\mathrm{ir})}$ are current densities contributed by the reversible and irreversible domain motions, respectively. The two solid-line fittings of the data show that Eq. (S1) works well for these cases. After N~50, the current becomes nearly invariant with a further increase in N. It was determined in this study that ~50 cycles are required to remove the contribution from $\Delta P(V_f)$ at each V_f. Therefore, for the measurements of C_f- V_f loops in the main text of Figs. 3 (a)-3(b), the capacitor was pre-poled at -6 V for 600 ns, and the following measurements were made: at each V, the capacitor was first stressed for 70 cycles, which must be sufficient to remove the irreversible polarization at V invoked by the increased step voltage, and $\Delta Q_f(V)$ was estimated as described below.

Fig. S2b shows an example of how the reversible capacitor charge was estimated. The blue line in Fig. S2b shows the first measurement of the reversible capacitor charging current within 300 ns at V = 0.5 V after cycling under a unipolar pulse with a voltage/width of 0.5

V/300 ns for 70 cycles. Next, the red line shows the subsequent measurement by suddenly changing V from 0.5 to 0.45 V after capacitor charging time of 120 ns. The inset in Fig. S2b shows the difference in the two currents. The integration of this difference corresponds to the reversible domain polarization charge of $\Delta Q_f(V)$ within ΔV over the time of 180 ns, where ΔV = -0.05 V in this case. Meanwhile, the voltages sensed by the ferroelectric layer at V and ΔV were $V_f = V - JSR$ and $\Delta V_f = \Delta V - \Delta JSR$, respectively, where ΔJ is the current difference between V and $V+\Delta V$. With these relationships, C_f can be calculated as a function of V_f , i.e., $C_f(V_f) = \Delta Q_f(V_f)/\Delta V_f$. When ΔV_f is required to be constant at each V_f for the whole C_f - V_f characterization, for example, in the main text of Figs. 3a-3b, a self-adjusting computer program can be formulated to automatically search for the suitable ΔV value at each V_f with dynamical adjustment of $J(V_f)$.

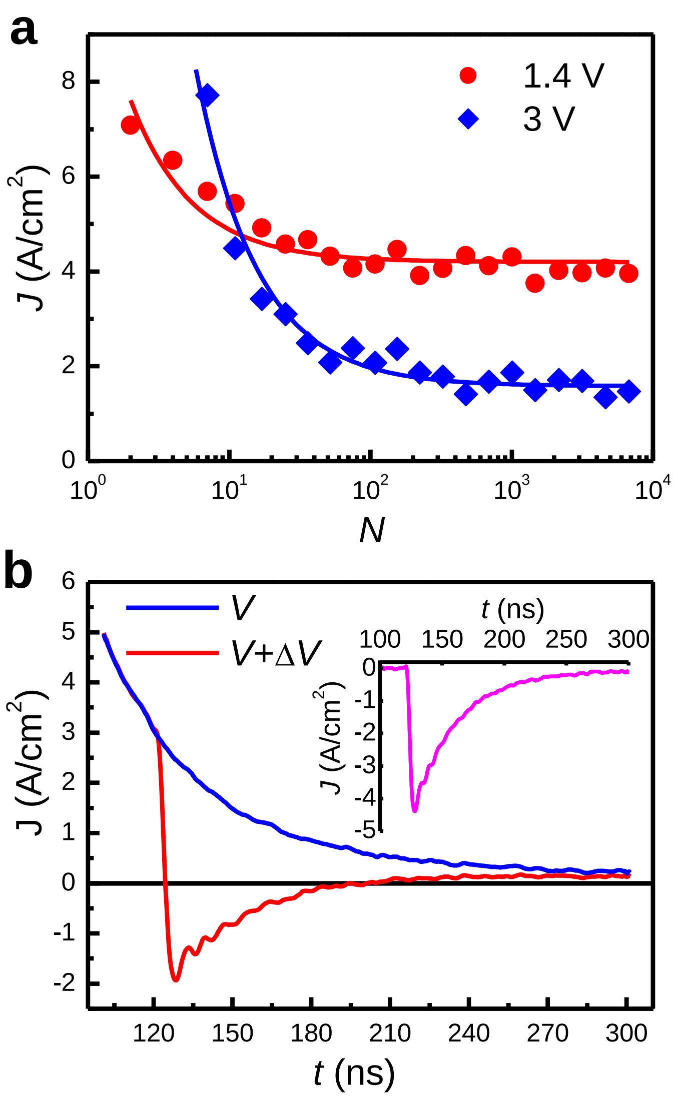

Figure S2 | Differential capacitance measurement from short pulses. (a) Dependence of the capacitor charging current density at 250 ns on the cyclic number for an oppositely polarized 10^4-μm^2-area Pt/PZT/Pt capacitor under different unipolar pulse voltages with a 250 ns width in a 2 MHz frequency, where the solid lines are the best fits of the data in accordance with Eq. (S1). (b) After unipolar pulse stressing with a voltage/width of 0.5 V/300 ns for 70 cycles, the reversible capacitor charging current was first measured within 300 ns at V = 0.5 V. This measurement was then repeated by suddenly superimposing ΔV = -0.05 V above V after a capacitor charging time of 120 ns. The inset shows the difference between the two currents.

After mathematically smoothing the $P_{nu}(V_f)$ and $P_d^{(P)}\left(V_f\right)\Big|_{V=8\mathrm{V}}$ curves in the main text of Figs. 4b-4d, the nonlinear capacitance $C_f = -\frac{dP_{nu}}{dV_f} - \frac{dP_d^{(P)}}{dV_f}$ was calculated directly from the capacitor discharging, and the results are shown in Fig. S3 for different V (Fig. S3 in the left panel, S = 100×100 μm^2) and S (Fig. S3 in the right panel, V = 3.5 and 8 V for P_{nu} and $P_d^{(P)}$, respectively) values. During this calculation, the formulae of $P_{nu}\left(V_f\right) = A\left[\tan\left(BV_f^2 - C\right) + \tan C\right]$ and $P_d^{(P)}\left(V_f\right) = \sum_{n=0}^{5} k_n V_f^n$ were adopted to smooth the $P_{nu}(V_f)$ and $P_d^{(P)}(V_f)$ plots, respectively, where A, B, C and k_n are fitting parameters. From the two formulae, the total capacitance per area below the coercive voltage for domain nucleation, $V_{c,n}$, as well as the individual capacitance from $C_f^{(P)} = -\frac{dP_d^{(P)}}{dV_f}$ were calculated. In principle, $C_f(V_f)\Big|_{V_f = V_c^n} \to \infty$ if the distribution in $P_{nu}(V_f)$ is extremely narrow near V_f = $V_{c,n}$, as represented by the dashed lines in Fig. S3. Experimentally, however, C_f has “only” a ~tenfold enhancement within the 0.2 V range near $V_{c,n}$ due to the finite distribution in P_{nu} with respect to V_f.

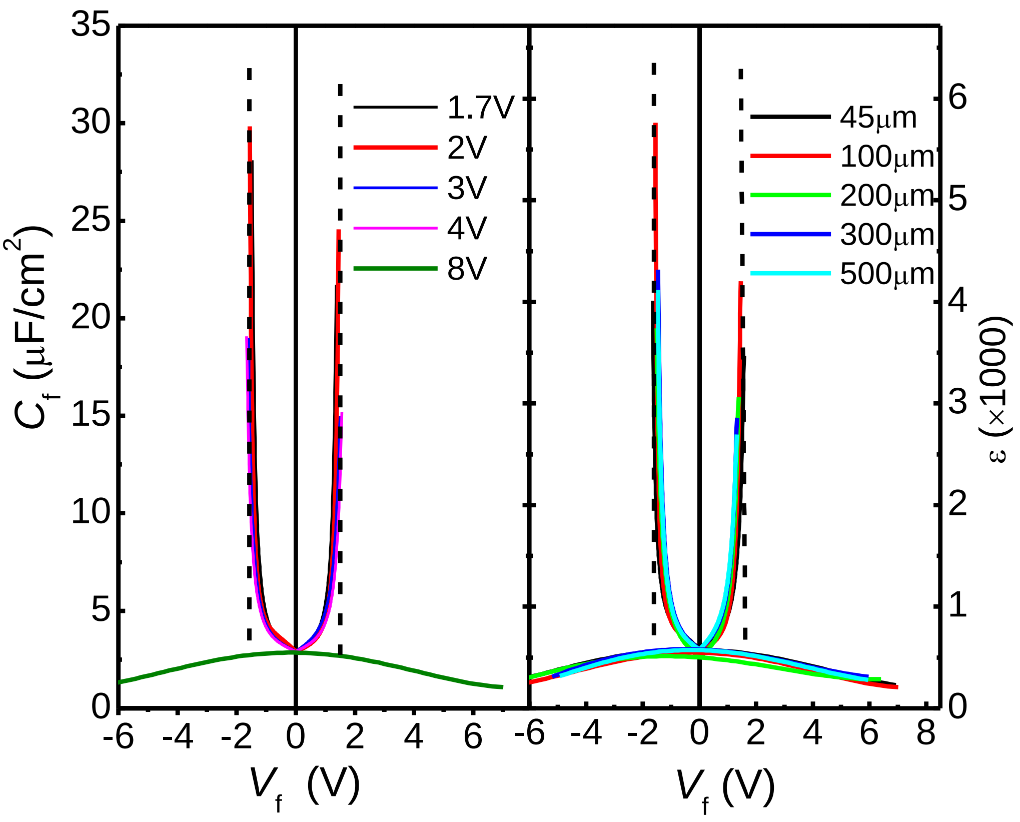

Figure S3 | Calculated nonlinear capacitance. The nonlinear C_f-V_f loops were calculated from the $P_d^{(P)}\left(V_f\right)\big|_{V=8\mathrm{V}}$ and $P_{\mathrm{nu}}(V_f)$ plots in the main text of Figs. 4b-4d, under different V values for a 10^4-μm^2-area capacitor (left panel), and with various capacitor sizes under V = 3.5 V (right panel).

Finally, the capacitance measured using the proposed pulse switching technique was compared with that from the conventional double "T" electric bridge that uses a small signal sine wave overlapped to V_f using a commercial HP 4194A impedance analyzer. The open symbols in Fig. S4a show the C_f - V_f loop from the pulse switching characterization under the voltage stressing time of 100 ms, with ΔV_f = -0.05 V. This loop complies with that shown by the solid line obtained from the commercial impedance analyzer using the same stressing time, with an AC oscillation amplitude of 0.05 V at a 1 MHz frequency, even though a slight misfit

occurred, especially near $V_{c,n}$. This tiny misfit was due to the accumulative voltage stressing time effect on the capacitance that appears in the commercial impedance analyzer during continuous voltage sweeping. This accumulative time effect is avoided, however, in the pulse switching technique, where the film is fully relaxed at zero voltage for ~5 s after each intermittent measurement at V_f. The dielectric loss from the measurement of the impedance bridge is in the range of 0.0076-0.032, as shown in Fig. S4a. However, this loss cannot be estimated from the pulse-type measurement due to the suggested current integration method.

As the P_{nu} distribution over V_f near $V_{c,n}$ is quite narrow (full-width half-maximum in the $P_{nu}(V_f)$ peak of ~0.3 V) in the aged Pt/PZT/Pt capacitors, the large $|\Delta V_f|$ value would smear out the sharpness of dP_{nu}/dV_f and would thus reduce ε of Figs. 3a-3b in the main text. This ε reduction is different from the decrease in ε by the suppression of the domain wall vibrations being ascribed to the pinning of the domain wall to the defects in most ferroelectrics, where ε actually increases with the increasing $|\Delta V_f|$, as shown in Fig. S4b. Generally, this ε increase by the increased wall vibrations is linear with $|\Delta V_f|$ and is described by the Rayleigh law [S1]. In the soft ferroelectrics, however, such as the PZT thin film, where the pinning centers are not randomly distributed, this dependence obeys a power law, as shown by the solid-line fitting of the data in the inset of Fig. S4b [S2]. Fig. S4c shows the two types of nonlinear dependences of Q_f on $V_f[Q_f(V_f)]$. For the type I to match Figs. 4c-4d in the main text, the nonlinear dielectric capacitance $C_f(V_f)=\Delta Q_f(V_f)/\Delta V_f$ at the point **P** increases as $|\Delta V_f|$ reduces from $|\Delta V_{f1}|$ to $|\Delta V_{f2}|$, in agreement with the measurements in the main text of Figs. 3a-3b. In contrast, for the type II in Fig. S4b, which can be described by the Rayleigh law, $C_f(V_f)$ decreases as $|\Delta V_f|$ reduces from $|\Delta V_{f1}|$ to $|\Delta V_{f2}|$.

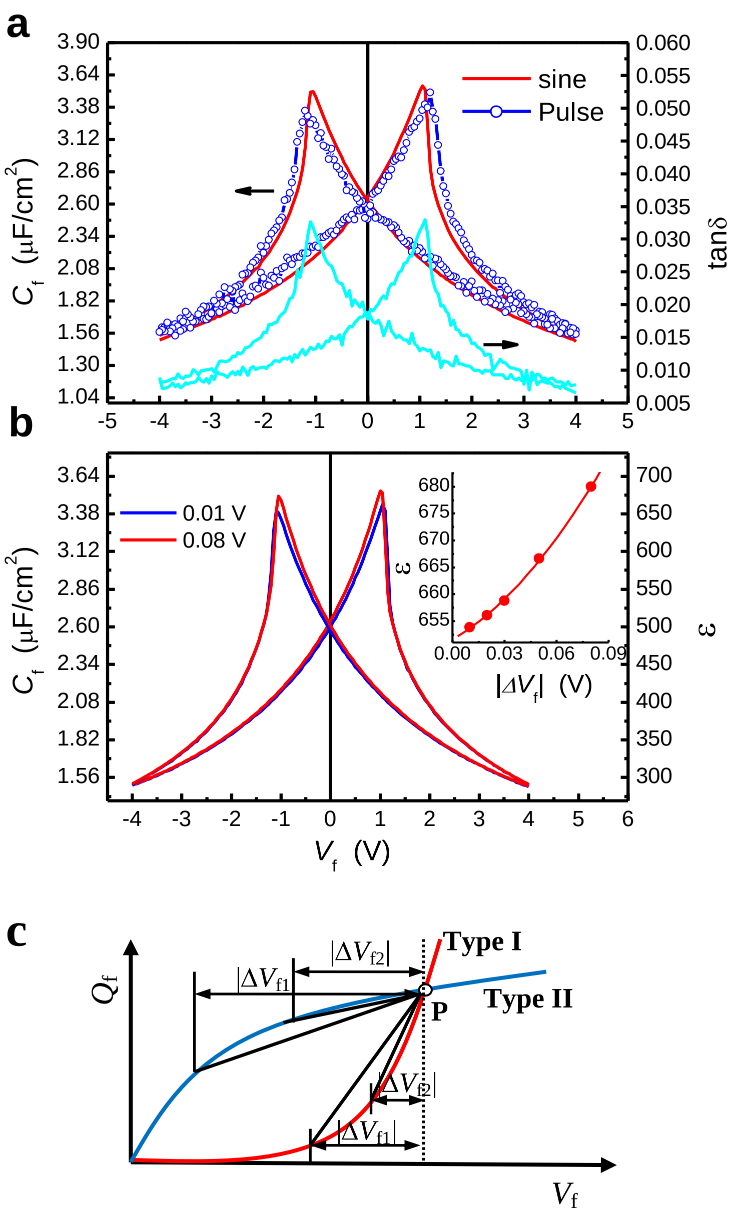

Figure S4 | Nonlinear capacitance comparison. (a) The open symbols show the capacitance-voltage loop from the pulse measurement induced by ΔV_f = -0.05 V under a voltage stressing time of 100 ms compared to the solid-line loop measured from the commercial HP 4194A impedance analyzer with an AC amplitude of 0.05 V at 1 MHz. (b) Capacitance-voltage loop from the commercial HP 4194A impedance analyzer with different amplitudes $|\Delta V_f|$ at 1 MHz. The closed symbols in the inset show the dielectric enhancement with increasing voltage amplitude at V_f = 1 V, where the solid line is the fit of the data according to the power law [S2]. (c) The schematic demonstration of nonlinear capacitance variation at the point **P** with $|\Delta V_f|$ from $|\Delta V_{f1}|$ to $|\Delta V_{f2}|$ in two types of nonlinear $Q_f(V_f)$ dependences.

In order to control the domain oscillation within the film thickness, the voltage stressing time must be shorter than the domain forward growing time Δt in the main text of Fig. 2. From the conventional technique using the impedance analyzer, the maximum measuring frequency is usually much smaller than the reverse of the circuit RC time constant. The RC time constant could be very large when $V \rightarrow V_{c,n}$ (if $C_f \rightarrow \infty$, $RC_f \rightarrow \infty$). In this sense, the impedance analyzer technique is unsuitable to measure the short-time capacitance to match with the fast domain longitudinal oscillation speed.

However, the delta pulse technique in Fig. S2b can resolve this problem. Theoretically, from the short-time current integrations across R in the circuit under V and $V+\Delta V$, we have

$$\Delta Q_f\left(V_f\right) = Q_f\left(V+\Delta V\right) - Q_f\left(V\right) = \int_0^{\Delta t} \frac{V+\Delta V}{R} e^{-t/RC_f} - \int_0^{\Delta t} \frac{V}{R} e^{-t/RC_f} = \Delta V C_f\left(1-e^{-\Delta t/RC_f}\right). \quad \text{(S2)}$$

At this moment, the voltage change across C_f is

$$\Delta V_f(\Delta t) = \left(V+\Delta V\right)\left(1-e^{-\Delta t/RC_f}\right) - V\left(1-e^{-\Delta t/RC_f}\right) = \Delta V\left(1-e^{-\Delta t/RC_f}\right). \quad \text{(S3)}$$

From the above two equations, we have $\Delta Q_f(V_f)/\Delta V_f = C_f$, which is independent of Δt.

Figs. S5a-5d show the results using this delta pulse technique for a commercial linear capacitor with C_f = 2.2 nF. Fig. S5a shows the capacitor charging current transient $I(t)$ at V = -4.6 V as well as the discharging current transient induced by ΔV with ΔV_f = -0.05 V after voltage stressing time of t = 0.8 μs, where the inset shows the current difference of the two current transients. From the equation of $C_f(V_f)=\Delta Q_f(V_f)/\Delta V_f$, we got corresponding C_f-V_f loops under different ΔV_f in Fig. S5b. All the data overlap together in the plots, although V and ΔV are not fully dropped across C_f during electrical characterization time with t = 1 μs and Δt = 200 ns (i.e., $V \neq V_f$, $\Delta V \neq \Delta V_f$). Later, we repeated this measurement with t = 40 μs and Δt =200 ns in Fig. S5c, where V is fully applied over C_f ($V = V_f$, $\Delta V \neq \Delta V_f$). The corresponding C_f-V_f loop was plotted in Fig. S5d, which is the same as those in Fig. S5b. If we further increase Δt from 200 to 500 ns in Fig. S5d so that $V = V_f$ and $\Delta V \cong \Delta V_f$, the two loops still overlap together.

Finally, we compared these plots to the data adopted from a commercial HP 4194A impedance analyzer using a sine wave at 100 kHz, and the agreement is obvious in Fig. S5d.

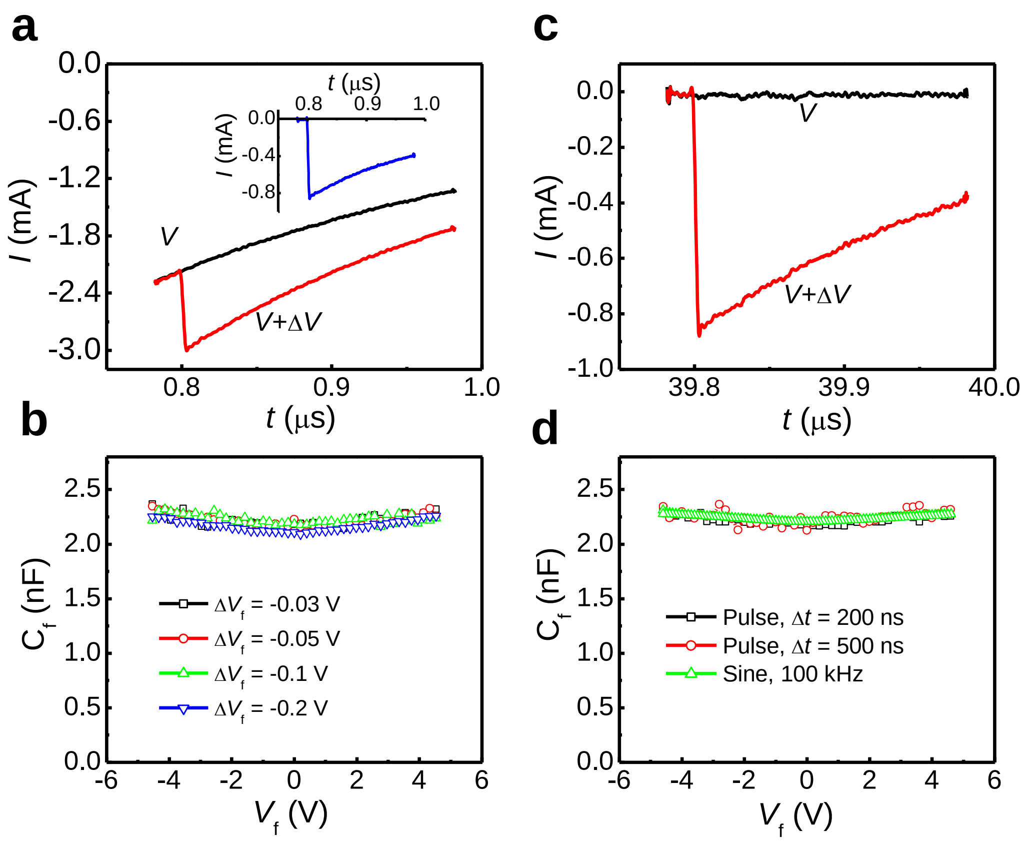

Figure S5 | The short-time capacitance from a pulse-type technique for a standard linear capacitor with C_f = 2.2 nF. (a) The capacitor charging current transient under V = -4.6 V as well as the discharging current transient induced by ΔV with ΔV_f = -0.05 V after voltage stressing time of 0.8 μs, where the inset shows the current difference of the two current transients. (b) C_f-V_f loops under different ΔV_f from the pulse technique in (a). (c) The capacitor charging current transient under V = -4.6 V as well as the discharging current transient induced by ΔV with ΔV_f = -0.05 V after voltage stressing time of 39.8 μs. (d) C_f-V_f loops with ΔV_f = -0.05 V under different adoption times (Δt) from the oscilloscope using a short-time pulse technique in (c), in comparison to the data from a commercial HP 4194A impedance analyzer using a sine wave at 100 kHz.

B. Domain Nucleation and Sideways Wall Motion

In the main text of Fig. 4a, domain switching currents under a series of pulses with the step-by-step increase in the pulse width from 5 to 500 ns were shown by keeping these pulse voltage at V = 4 V for a 10^4-μm^2-area Pt/PZT/Pt capacitor. Later, the capacitor discharge density of either $P_d^{(U)}(V_f)\big|_{V=4\mathrm{V}}$ or $P_d^{(P)}(V_f)\big|_{V=4\mathrm{V}}$ was obtained in Fig. 4b as a function of V_f from the capacitor discharging current integration for the pre-poled domains with their orientation antiparallel or parallel to the applied field. With this technique, the nucleating domain relaxation after domain switching pulse width can be understood from the appearance of a sharp discharge peak at $V_{c,n}$ in the $P_d^{(U)}$-V_f plot, which nevertheless disappears from the $P_d^{(P)}$-V_f plot. For the better understanding of the nucleating domain mechanism after these switching pulses, corresponding P_f-V_f hysteresis loops are shown in Fig. S6c under bipolar pulses shown in Fig. S6a with the step-by-step increase in the first pulse width from 5 to 300 ns, and their domain switching current transients are shown in Fig. S6b.

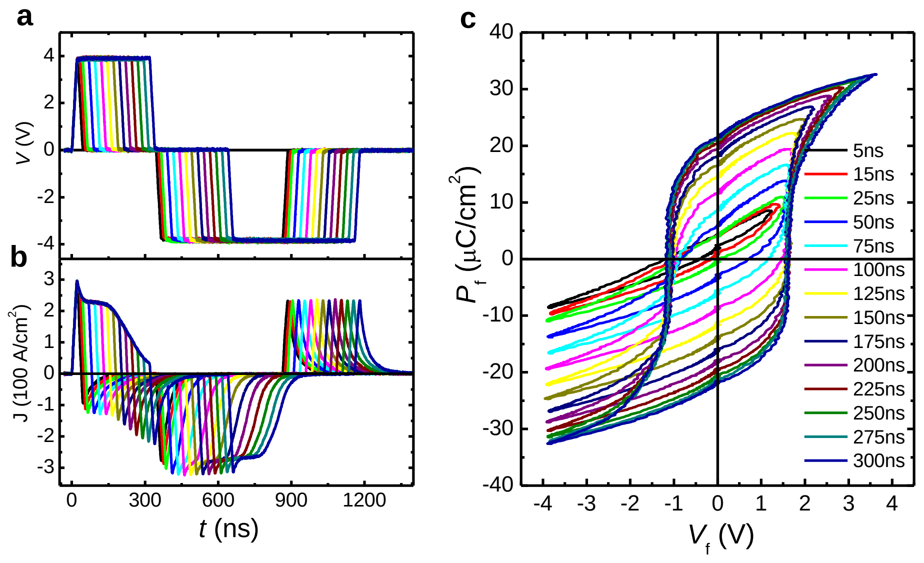

Figure S6 | Doman switching currents and P_f-V_f hysteresis loops under step-by-step increase in pulse width. (a) Input bipolar pulses separated at 300 ns with step-by-step increase in the first switching pulse width from 5 to 300 ns by fixing the second presetting pulse width at 500 ns. (b) Domain switching current transients under the above bipolar pulses for a 10^4-μm^2-area Pt/PZT/Pt capacitor. (c) P_f-V_f hysteresis loops inferred from domain switching current transients.

To narrow down the $P_{nu}(V_f)$ or $\Delta P(V_f)$ distribution near $V_{c,n}$, all the samples in the main text were first stressed at ±6 V with appropriate bipolar pulse widths for 10^4 times. The samples were then aged at ambient temperature for more than one week, for the establishment of the same imprint history for all the domains that initially had random orientations. The V_f range for inducing the change in ΔP from 0 to its maximum was as high as ~0.6 V for the 10^4-μm^2-area Pt/PZT/Pt capacitor when it was not treated as mentioned above (Fig. S7a). This value decreased to ~0.3 V when the sample was treated, as shown in Figs. 2c-2d of the main text. If the $\Delta P(V_f)$ distribution was wide (0.6 V), the C_f - V_f peak was broadened, as can be seen in Fig. S7a, which resulted in ~42% decreases in the maximum capacitance, in comparison to the C_f - V_f loop for the aged capacitor in Fig. S7c.

The peak position in the C_f - V_f loop corresponds to the coercive voltage $V_{c,n}$ for the reversible domain nucleation and (partial) growth. This value is smaller than the average coercive voltage $V_{c,s}$ for the wall sideways motion, which can be estimated as the voltage that corresponds to ΔP = 22 $\mu C/cm^2$, as shown in Fig. S7a. After transforming the bipolar domain switching current into polarization-voltage hysteresis loops in Fig. S7b, the average coercive voltages $V_{c,s}$ for the wall sideways motion were achieved.

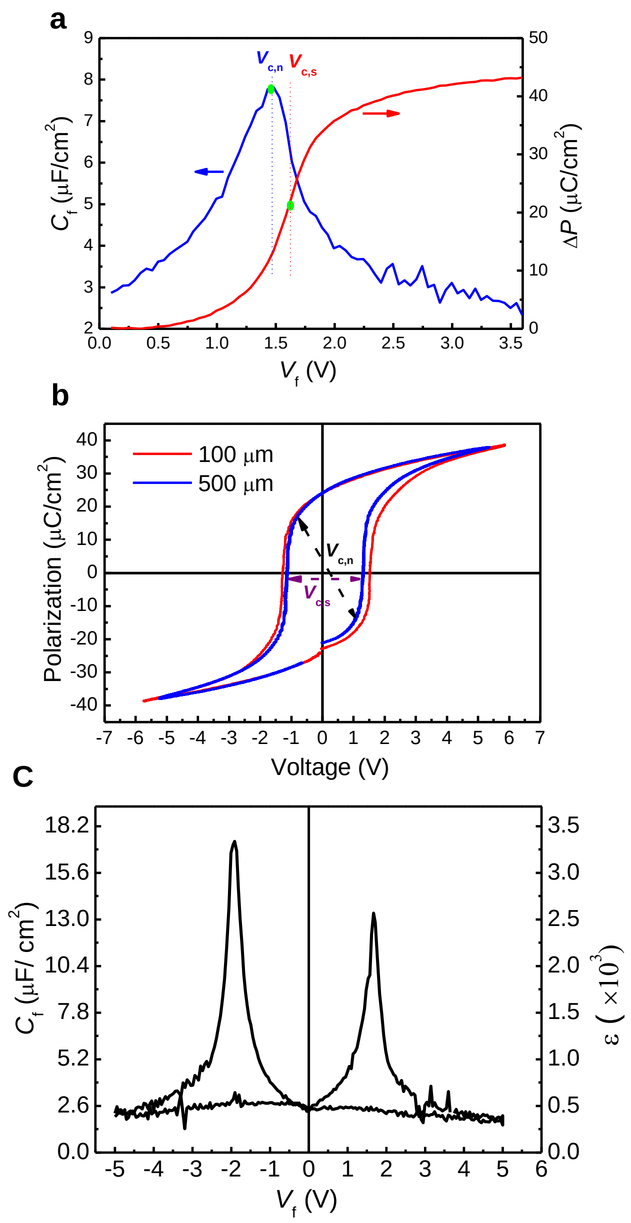

Figure S7 | Polarization distribution and coercive voltages. (a) C_f - V_f loops for a 10^4-μm^2-area fresh capacitor with a broad $\Delta P(V_f)$ distribution under ΔV_f = -0.05 V and a stressing time of 300 ns, where the domain nucleation voltage $V_{c,n}$ was different from the wall sideways motion voltage $V_{c,s}$. (b) Polarization-voltage hysteresis loops transferred from bipolar domain switching current transients under pulses with voltages/widths of ±6 V/300 ns and ±5.5 V/8000 ns for capacitors with 100 and 500 μm side lengths, respectively. (c) C_f - V_f loops for a 100 × 100 μm^2 capacitor which was aged under ΔV_f = -0.05 V and a stressing time of 300 ns.

C. Cryogenic Domain Nucleation and Imprint

A cross-section transmission electron microscopy (TEM) image in Figs. S8a-8b shows that the PZT film has a smooth surface and pinholes-free morphology. The average grain size was ~800 nm, which is far larger than the film thickness of 130 nm. The large grain size could reduce the inhomogeneity of domain nucleation due to the lower contribution of regions influenced by grain boundaries. As the polarization reversal initiates from the oppositely polarized nuclei at the defects, such as dislocations or inclusions with low nucleation energies at the film interface[S3], it is necessary to study the influences of the interface nature and temperature on domain nucleation. For this purpose, an IrO_2/Pt top electrode was adopted for a 130-nm-thick PZT thin-film capacitor with an area of 100×100 μm^2. Oxide electrodes are known to be usually fatigue-resistive [S4].

After cooling the sample down to 77.6 K, a series of voltage pulses with different widths were applied to the top electrode, and the discharging charge densities $P_d^{(P)}$ and $P_d^{(U)}$ were measured as functions of voltage V_f across the PZT film as Figs. 4a-4d of main text. The results are shown in Fig. S8c. After domain switching, the discharge density under V = 6 V decays continually with the broadened stressing pulse width, as shown in Fig. S8d. The measurement protocols are identical to the case of the Pt/PZT/Pt capacitor described in the main text, and Fig. S8e shows the extracted P_{nu} for the V values of 3, 5, and 8 V.

All the polarizations of the nucleating domains had sharp peaks near $V_{c,n}$, with $|P_{nu}|$ = 4.4 $\mu C/cm^2$ and an interfacial nucleus density of 7.0 μm^{-2}, which are slightly higher than 4.1 $\mu C/cm^2$ and 3.8 μm^{-2} in Pt/PZT/Pt capacitors at ambient temperature. From the calculated C_f-V_f loop in Fig. S8f, maximum dielectric constant at 77.6 K was higher than 7000. Therefore, it can be understood in this study that the types of electrodes and measurement temperature have weak influence on reverse domain nucleus density and their reversible motion.

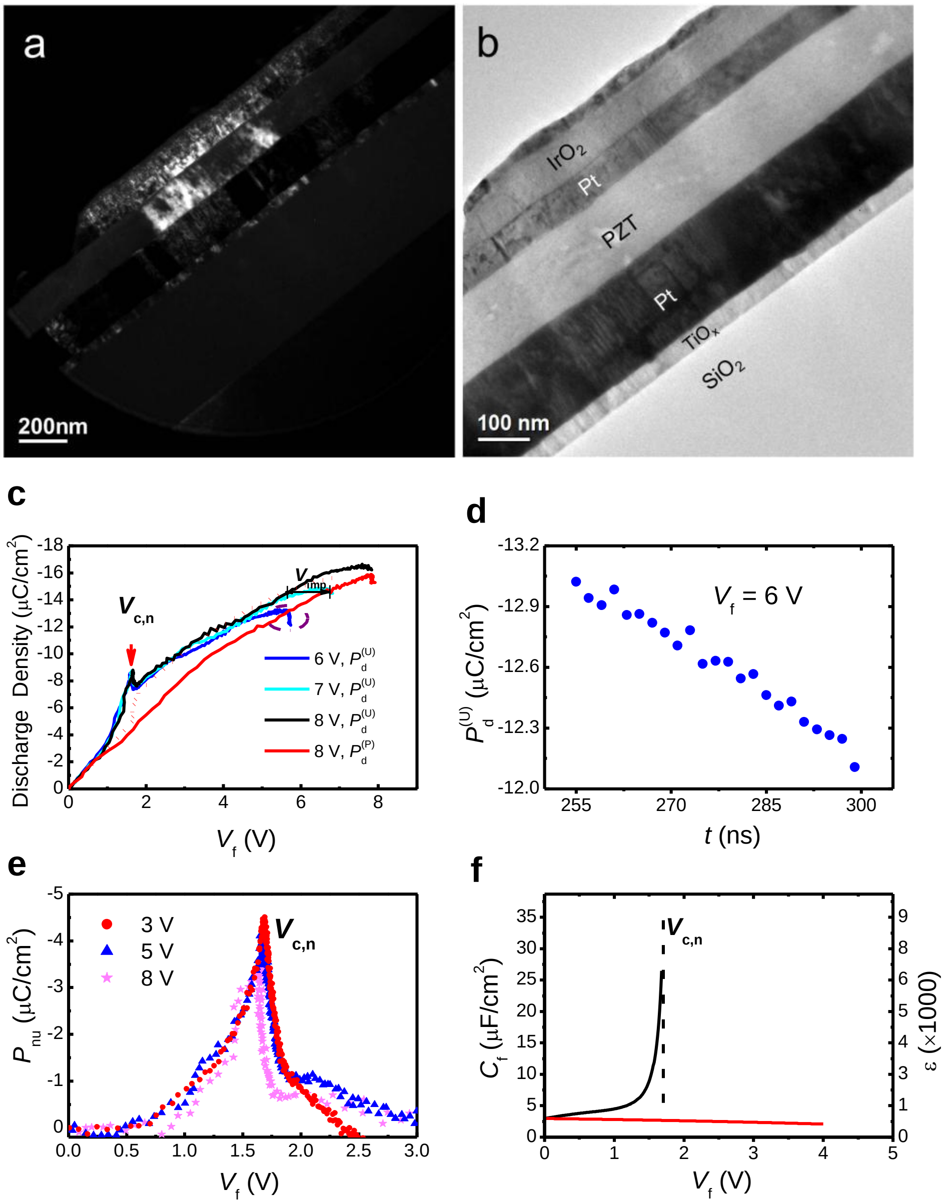

Figure S8 | Reversible and irreversible polarizations at 77.6 K. (a) The dark filed, and (b) bright field TEM images showing the stacked structure of the PZT capacitor. **(**c) Voltage dependence of the discharging charge density for a 10^4-μm^2-area IrO_2/PZT/IrO_2 capacitor under different V values, with E_f parallel/anti-parallel to the pre-poling polarization P_f, where the dotted line shows the imprint effect on shift $P_d^{(P)}\left(V_f\right)\Big|_{V=8\text{V}}$ along with the irreversible polarization reversal. (d) The inset shows the $|P_d^{(U)}|$ degradation with the prolonged voltage stressing time at $V_f = 6$ V. (e) The separated reversible nucleating domain polarizations under different V values. (f) The calculated capacitance per area from the two formulae of $C_f = -\frac{dP_{nu}}{dV_f} - \frac{dP_d^{(P)}}{dV_f}$ (the black solid line) and $C_f^{(P)} = -\frac{dP_d^{(P)}}{dV_f}$ (the red solid line), respectively.

D. AC Amplitude and System Noise

When the $|\Delta V_f|$ value was further decreased to 0.01 and 0.02 V as in the Fig. 3b of main text for the small capacitor with area of 2×10^3 μm^2, the characterization of the giant nonlinear capacitance at a small stimulating voltage becomes very difficult due to weakened voltage signals sensed by the oscilloscope as well as relatively higher system noise, as shown in Fig. S9c. When the applied delta voltage ΔV is maintained constantly at -0.05 V, the maximum absolute voltage of $|\Delta V_R|$ across R sensed by the oscilloscope decays quickly from 0.039 to 0.012 V with the decreasing capacitor area from 10^4 μm^2 to 10^3 μm^2. This $|V_R|$ reduction is due to the decreased circuit RC time constant under a finite rising/failing time t_r of the input pulse, which can be described by the equation

$$\Delta V_R = \frac{\Delta V}{t_r} RC_f \left[1 - \exp\left(-\frac{t_r}{RC_f}\right)\right]. \qquad \text{(S4)}$$

If $t_r \rightarrow 0$, $\Delta V_R = \Delta V$. For the Agilent 81150A arbitrary-pulse generator, the nominal t_r value provided by the manufacturer is supposed to be 2.5 ns; however, the measurement data in Figs. S9a-9b showed that this time is actually as long as 6 ns. This is the reason why the maximum $|\Delta V_R|$ signal decays so quickly with the reduction of the capacitor area in Fig. S9c, especially for the smallest 10^3 μm^2-area capacitor with the signal nearly merging into the system noises under $|\Delta V|$<0.05 V. On the other hand, the nonlinear giant dielectric permittivity usually appears at $V_{c,n}$ under a small stimulating voltage of $|\Delta V_f|$~ 0.01 V in the main text of Figs. 3a. Due to the limitation of the present system, we cannot exhibit the giant dielectric response of nucleating domain oscillation for a small capacitor with the area of 2×10^3 μm^2 in the main text of Fig. 3b. Expectedly, the characteristic frequency of this giant dielectric response can be adjusted in by changing either S or R according to Eq. (2) in the main text.

If the polarity of applied voltages is the same to the pre-poling direction of the domains, the circuit RC time constant is ~37.2 ns for the 10^4 μm^2-area capacitor in the main text of Fig.

3a, far smaller than the dc voltage stressing time of 250 ns, as shown in Fig. S9a. This time constant can be reduced down to 9.2 ns in Fig. S9b if the capacitor area is shrunk down to 2×10^3 μm^2. Under this condition, $V \cong V_f$ and $\Delta V \cong \Delta V_f$. In contrast, as $V_f \rightarrow V_{c,n}$, the dielectric constant has more than 16-fold enhancement with the applied voltages being opposite to the pre-poling direction. In this case, the circuit *RC* time constant accordingly increases to ~600 ns, and $V \neq V_f$ and $\Delta V \neq \Delta V_f$. Under this circumstance, the pulse width has to be increased to such time scale to make $V \cong V_f$ and $\Delta V \cong \Delta V_f$. However, the reverse nucleating domain should grow up to the opposite electrode and the sideways domain motion may dominate through the whole film thickness during such a long pulse time, which is a situation that has to be strictly prohibited for the purpose of this work. In this sense, what we have presented in this work is a new technique to characterize C_f within a limited time scale even when $V \neq V_f$ and $\Delta V \neq \Delta V_f$. Therefore, the development of a short-time delta pulse technique in Supplementary part A in consideration of non-zero current correction is very necessary to measure the reversible displacement charge only with $\Delta V < 0$, which is immune to the disturbance of the irreversible remanent polarization charge. This experimental setup is well calibrated using a standard linear (non-ferroelectric) capacitor with C_f = 2.2 nF in Figs. S5a-5d. Moreover, the highest dielectric response in main text of Figs. 3a-3b always occurs at around $V_{c,n}$, which sufficiently rules out of any calculation artifacts arising from $V \neq V_f$ and $\Delta V \neq \Delta V_f$.. Otherwise, the artifacts should be magnified with the enhancing capacitance charging/discharging current which would be maximum at +/-5 V in the main text of Figs. 3a-3b. However, this never comes true, since we always observed nearly overlapping C_f-V_f curves independent of ΔV_f at such high voltage regions in lack of nucleating domain contribution.

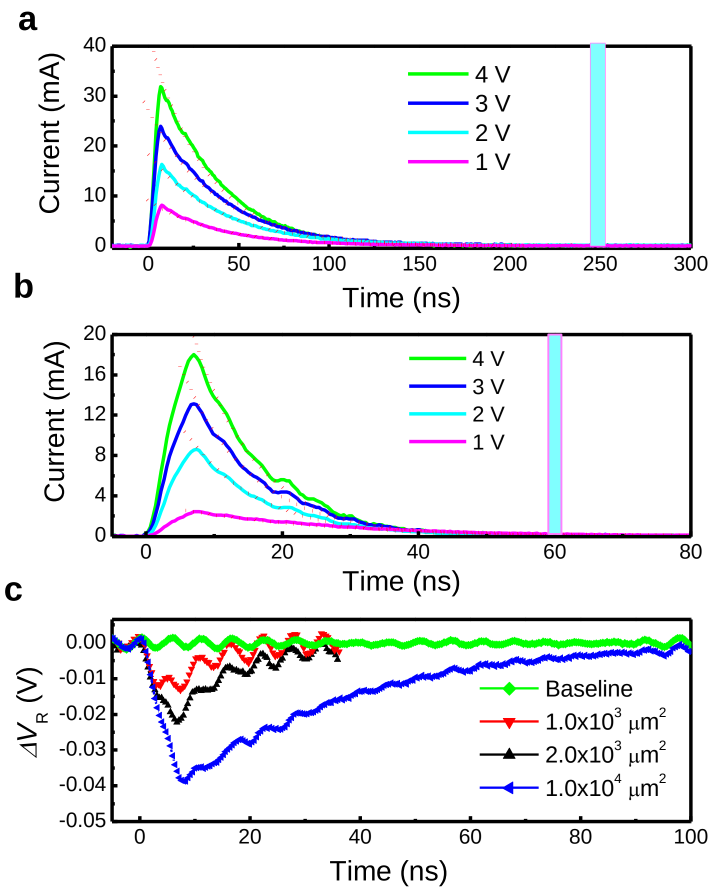

Figure S9 | Non-switching capacitor charging currents under different applied voltages for the capacitors with areas of (a) 10^4 μm^2 and (b) 2×10^3 μm^2 with pre-poling domain direction parallel to the voltage polarity, where the colored rectangular bars show the times to measure the capacitance for the estimation of nucleating domain oscillation under the fields. (c) Voltage transients with time across the total resistors of R = 100 Ω in-series with a ferroelectric capacitor in different electrode areas in the circuit sensed by the oscilloscope under ΔV = -0.05 V.

E. Film Topography

Scanning electron microscopy (SEM) photograph from a planar view in Fig. S10a shows the PZT films which have a smooth surface free of the pinholes. The average grain size is around 850 nm, which is far larger than the film thickness of 170 nm, as shown from a cross-sectional view in Fig. S10b. The large grain size could reduce the inhomogeneity of domain nucleation at regions around gains and grain boundaries.

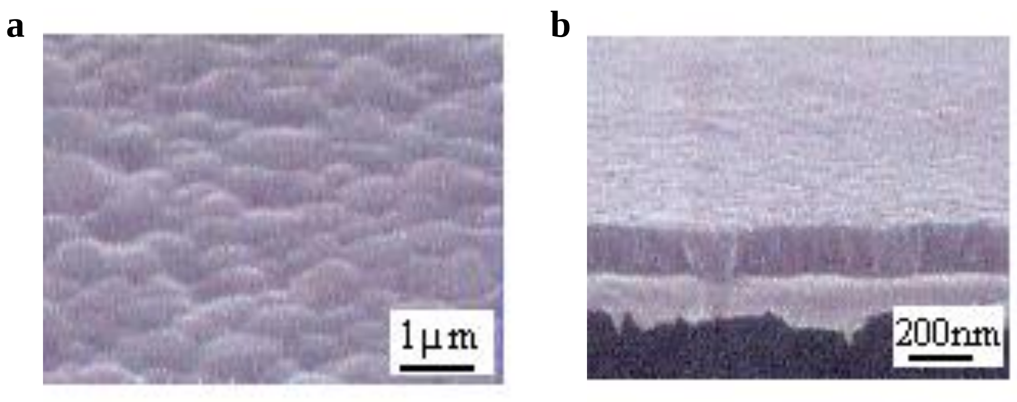

Figure S10 | The film topography. The grain size and film thickness for PZT were inferred from SEM photographs of **a**, in a planar view and **b**, in a cross-sectional view.

F. Synchronization of domain nucleation and pulse application

Figure S11a shows voltage dependence of dielectric permittivity under different voltage sweeping times. The dielectric permittivity increases rapidly as the sweeping time is shortened to match domain nucleation time at around $V_{c,n}$. Figure 11b shows the pulse-time dependence of dielectric permittivity under different stressing voltages across the ferroelectric film only near $V_{c,n}$. The dielectric permittivity shows a broad peak near the pulse-time of 275 ns when the voltage was 1.6 V, and the peak becomes sharper and shifts toward to a lower value of time with the increasing voltage. The peak locates at 204 ns when the voltage was 1.9 V. Such peaking behavior is due to the synchronization of ΔV_f stimulating time to the voltage-dependent domain nucleation time, which constitutes the major reason for the generation of a giant dielectric response described by Fig. 1 in the main text.

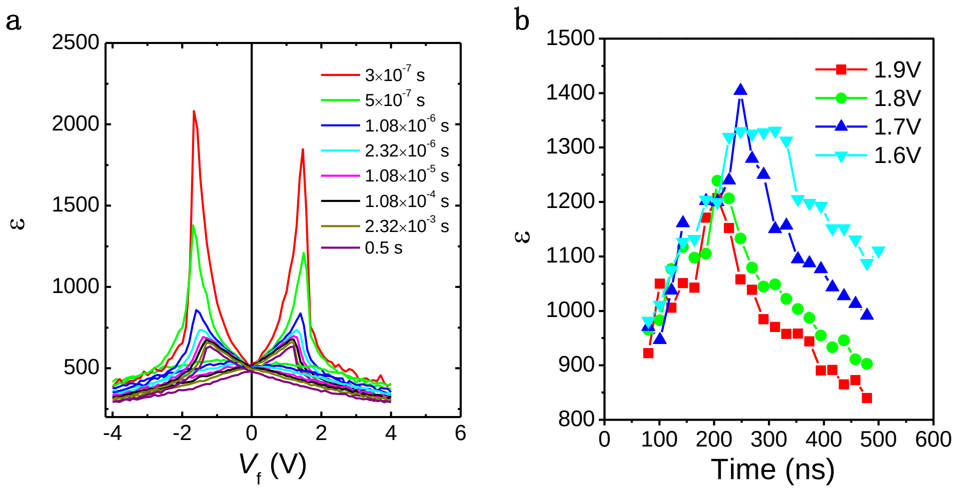

Figure S11 | Time and voltage dependences of dielectric permittivity. **a**, Voltage dependence of dielectric permittivity under ΔV_f = -0.1 V with the prolong voltage stressing time from 300 ns to 0.5 s for the 10^{-4} μm^2-area PZT capacitor in series with a resistor of R = 100 Ω in the circuit. **b**, Pulse-time dependence of dielectric permittivity with ΔV_f = -0.1 V under various stressing voltages.

G. References

S1 Griggio, F., *et al*. Substrate clamping effects on irreversible domain wall dynamics in lead zirconate titanate thin films. *Phys. Rev. Lett*. **108**, 157604 (2012).

S2 Borderon, C., Renoud, R., Ragheb, M. & Gundel, H. W. Description of the low field nonlinear dielectric properties of ferroelectric and multiferroic materials. *Appl. Phys. Lett.* **98**, 112903 (2011).

S3 Nelson, C. T., *et al*. Domain dynamics during ferroelectric switching. *Science* **18**, 968-971 (2011).

S4 Nagaraj, B., Aggarwal, S. & Ramesh, R. Influence of contact electrodes on leakage characteristics in ferroelectric thin films. *J. Appl. Phys.* **90**, 375 (2001).

H. Table I List of Parameters

Quantity	Definition	units
V	Externally applied voltage	V
V_f	Voltage drop across the ferroelectric film	V
V_{imp}	Imprint voltage	V
ΔV	Externally applied delta voltage	V
ΔV_f	Delta voltage sensed by the ferroelectric film	V
ΔV_{step}	Voltage steps to measure capacitance-voltage loops	V
S	Capacitor area	cm^2
R	Total in-series resistance	Ω
I	Current	mA
J	Current density	A/cm^2
J_n	Current density for domain nucleation	A/cm^2
J_s	Current density for wall sideways motion	A/cm^2
$P_d^{(U)}$	Discharge density for domains antiparallel to the field	$\mu C/cm^2$
$P_d^{(P)}$	Discharge density for domains parallel to the field	$\mu C/cm^2$
$2P_r$	Double remanent polzarization	$\mu C/cm^2$
P_s	Saturated polarization	$\mu C/cm^2$
ΔP	Switched polarization	$\mu C/cm^2$
P_{nu}	Nucleating domain polarization	$\mu C/cm^2$
P_f	Total polarization across ferroelectric films	$\mu C/cm^2$
$V_{c,n}$	Coercive voltage for domain nucleation	V
$V_{c,s}$	Coercive voltage for wall sideways motion	V
t	Time	ns
t_0	The moment to apply an opposite field	ns
τ	Duration of pulsed voltages	ns
Δt	Domain forward growing time	ns
t_n	Time for domain nucleation	ns
t_s	Time for domain wall sideways motion	ns
C_f	Capacitance per area for ferroelectric thin films	$\mu F/cm^2$
$C_f^{(nu)}$	Capacitance per area for nucleating domains	$\mu F/cm^2$
$C_f^{(P)}$	Capacitance per area for a normal capacitor	$\mu F/cm^2$
C_P	Parasitic capacitance in the circuit	$\mu F/cm^2$
Q_f	Charge density of ferroelectric capacitors	$\mu C/cm^2$
ΔQ_f	Delta charge density of ferroelectric capacitors	$\mu C/cm^2$
E_f	Applied field to ferroelectric capacitors	MV/cm
E_a	Domain activation field	MV/cm
E_c	Coercive field for domain switching	MV/cm
N	Number for cycles	
V_n	Nucleus volume	nm^{-3}
α	Inclined wall angle to the film surface	^{o}
D	Film thickness	nm

X	Volume fraction of the switched domains	%
ε_0	Permittivity in vacuum	F/m
ε	Dielectric constant	
t_r	Pulse rising/failing time	ns
V_R	Voltage across in-series resistors	V
